# Changes in oral health-related quality of life before and after dental treatment in 8–12-year-old Costa Rican schoolchildren

**DOI:** 10.3389/fdmed.2023.1167845

**Published:** 2023-04-27

**Authors:** Juliana Jiménez-Lobo, Daniela Batista-Cárdenas, Ariadna Aguilar-Cubillo, Adrián Gómez-Fernández, Karol Ramírez

**Affiliations:** ^1^ Faculty of Dentistry, University of Costa Rica, San José, Costa Rica; ^2^ School of Statistics, University of Costa Rica, San José, Costa Rica

**Keywords:** caries, dental treatment, hypomineralizations, malocclusions, oral health-related quality of life, quality of life, schoolchildren, sociodemographic factors

## Abstract

**Aims:**

The aims of this study were to (1) evaluate oral health-related quality of life (OHRQoL) in 8–12-year-old Costa Rican schoolchildren before and after dental treatment and (2) collect clinical and sociodemographic characteristics.

**Methods:**

Schoolchildren completed the Child Oral Health Impact Profile-Short Form (COHIP-SF-19) questionnaire before and after dental treatment. One of the parents was asked to complete a sociodemographic survey. Patients were treated for caries, hypomineralizations, and dental malocclusions. The prevalence of these conditions was assessed from the patient's electronic dental record.

**Results:**

Eighty participants (39 male and 41 female, average age: 9.4 ± 1.0 years) were recruited. The prevalence of dental caries was 56.1% with a mean deft and DMFT score of 3.15 ± 0.96 and 2.22 ± 0.77, respectively. The prevalence of hypomineralization and dental malocclusions was 53.7% and 82.9%, respectively. The Simplified Oral Hygiene Index before treatment was 1.45 ± 0.45 and after was 1.42 ± 0.43. The mean COHIP-SF-19 total score decreased from 53.7 ± 7.8 before dental treatment to 31.4 ± 4.2 after treatment. Improvements in all subdomains were also observed. Regarding sociodemographic characteristics, 65% of the patients lived in San José, Costa Rica's capital city, and 56.3% of the studied population belonged to a low-income family. Most parents did not complete high school. Regarding the number of family members living in the same house as the patient, an average of four people was reported. In relation to family structure, 58.8% of the children's parents lived together, either married or free union. As for household ownership, 53.8% of parents reported owning their house, 36.3% lived in a rented house, and 10% lived in a borrowed home.

**Conclusion:**

The prevalence of caries, hypomineralizations, and dental malocclusions were high before dental treatment. Reported sociodemographic characteristics unlikely changed after dental treatment, suggesting dental care played a pivotal role in improving self-perceptions of oral health and quality of life in our clinical setting.

## Introduction

Oral health-related quality of life (OHRQoL) describes an individual's perception of how oral diseases and conditions impact overall well-being (1, 2). OHRQoL captures consequences of good or poor oral health and aids clinicians and public health actors in identifying patients' concerns, expectations, and satisfaction with dental care received (3–5). The impact on quality of life of disease and treatment of disease and its outcomes should be considered when assessing health status and evaluating treatment results.

Several validated instruments currently exist to measure children's OHRQoL. The Child Oral Health Impact Profile (6) COHIP questionnaire is a self-administered, reliable, and interpretable instrument that captures positive impacts, such as confidence and attractiveness, and negative impacts like tooth pain (7). It has been translated and validated in many languages, including Spanish. The instrument appraises a condition-specific oral health impact on a child's daily life. Moreover, it can be used in an age range of 8–18 years old, differentiating children based on their clinical condition and clinical severity (8, 9). COHIP's long version consists of items comprising five subscales: oral health, functional well-being, social/emotional well-being, school environment, and self-image (10). Its short version, the Child Oral Health Impact Profile-Short Form 19 (COHIP-SF-19), can be administered more quickly and contains 19 items that evaluate the same subscales (11).

It is well known that caries, gingivitis, enamel defects, and malocclusions consistently reduce the quality of life of children and adolescents (6, 12–17). Dental caries still represents the most prevalent oral disease among children and adolescents worldwide and can affect quality of life through dental pain and lead to deterioration in oral function and emotional state (18–23). Furthermore, literature states hypomineralizations and dental malocclusions can affect quality of life of people since these conditions can affect aesthetics and functioning of teeth (24–28).

Although oral health access has improved significantly over the past decades, socioeconomic disparities remain among people living in poverty. Lower OHRQoL has been described among families facing greater socioeconomic disadvantages (29–31). In the same line, it has been proposed that sociodemographic factors such as age, gender, and education of parents are associated with OHRQoL in children (32). Children with educated parents and mothers more mature in age reported better OHRQoL (31). Ethnicity and race have been significantly associated with OHRQoL in children (33, 34), partly mediated through oral health status and how families and their children perceive oral health (35). Moreover, family structure has been found to influence psychological and psychosocial attributes and Oral Health-Related Quality of Life (OHRQoL) in children. For instance, children with only one adult in the household or living in overcrowded houses, with a large number of siblings, have been significantly associated with poor OHRQoL (29, 32, 36). Schoolchildren who attend private schools reported a better OHRQoL than those who attend public schools (29).

As advocates for increasing access to oral health care, The Faculty of Dentistry of the University of Costa Rica provides oral health treatments at a lower cost compared to private dental clinics. The sociodemographic profiles of pediatric patients seeking dental care at the Faculty of Dentistry have not yet been described. Children's perception about their OHRQoL and their clinical and sociodemographic profile is important to consider to update the individualization of interventions. Also, OHRQoL and sociodemographic data provide valuable information to clinicians in developing a child- and family-centered care approach.

So far, most studies investigating children's OHRQoL have been conducted in developed countries. Costa Rica is an upper middle-income and developing country. The nation has access to a highly rated healthcare system, public and private education, and access to potable water and electricity. All these factors combine to exclude the country from the category of being a less developed nation.

In Costa Rica, few studies have analyzed OHRQoL in adults and in the elderly (37–40). To the best of our knowledge, this is the first study evaluating OHRQoL in Costa Rican schoolchildren. Our objective was to determine the OHRQoL of 8–12-year-old schoolchildren pre- and post-dental treatment. Also, we wanted to assess the prevalence of oral conditions (caries, hypomineralizations, and dental malocclusions) and sociodemographic characteristics that have been associated with OHRQoL.

## Materials and methods

### Ethical aspects

This project was approved by the Ethical Scientific Committee of the University of Costa Rica (CEC-676-2021). Informed consent was obtained from one of the child's parents. Assent forms were also collected.

### Population of study

OHRQOL was determined in 8–12-year-old schoolchildren seeking dental care who attended the Clinic of Pediatric Dentistry or the Post-graduate Program of Pediatric Dentistry of the Faculty of Dentistry of the University of Costa Rica.

An appropriate sample size of 80 patients was calculated. This sample size allowed estimates with a confidence level of 95% and a maximum permissible error of 7% in the proportion of people with improved quality of life after dental treatment, which was estimated at 85%. The Finite Population Correction Factor was used. This sample was adjusted with a 10% non-response rate.

### Inclusion and exclusion criteria

Inclusion criteria for the study population were schoolchildren, aged 8–12 years old of both sexes, seeking dental care for caries, hypomineralizations, and dental malocclusions with no history of orthodontic treatment. Exclusion criteria were patients with cognitive impairment or other chronic illness, children with severe oral pain or limited range of motion of the jaws, and children under psychological treatment.

### Data collection

This study was conducted between the months of February 2022 and December 2022, in two phases. Phase one occurred during the month of February 2022, where all participants were recruited at their initial visit to the Faculty of Dentistry before dental treatment. Phase one included obtaining consent from one of the parents and a self-administered survey on socio-demographic data. Schoolchildren were asked to complete the Spanish version of the COHIP-SF-19 questionnaire. On that same day, a specialist in Pediatric Dentistry determined the children's oral health. When the initial visit was over, general information regarding oral conditions was obtained for each participant from the electronic dental record. Prevalence of caries, hypomineralizations, and malocclusions was collected. Information on missing and filled teeth was also extracted to report the deft/DMFT score. The simplified oral hygiene index (41) before treatment was annotated.

Phase two involved providing dental treatment to the participants. All participants received multiple treatments from March to December 2022. Participants received dental care for caries, hypomineralizations, and malocclusions. After completion of dental treatment, the COHIP-SF-19 questionnaire was reapplied to the participants one month after concluding the treatment. The response rate was 100 percent. The Simplified Oral Hygiene Index (41) was annotated again after treatment.

### Surveys

Collected socio-demographic data included age of the child, province of residence, household, household income, if parents live together or separated, mother and father's educational level, and home ownership (if the parent or guardian lived with their child in an owned home, a rented house, or a borrowed house).

To assess OHRQoL, the validated Spanish version of the COHIP-SF-19 was utilized. The COHIP-SF-19 questionnaire includes 19 questions, forming three conceptual subscales: oral health well-being; functional well-being; and socio-emotional well-being, school environment, and self-image. Schoolchildren were asked how often they had experienced oral impacts during the past 3 months. Each question could be answered using a Likert scale, ranging from “never” = 0, “almost never” = 1, “sometimes” = 2, “fairly often” = 3, to “almost all the time” = 4. Scoring of the positively worded items was reversed, while scoring of the negatively worded items was not.

The COHIP-SF-19 overall score was calculated by summing the scores for all 19 items within a range of 0–76. Thereby, higher scores reflected a more positive OHRQoL, and lower scores reflected an impacted oral health-related quality of life.

### Statistical analysis

Data was tabulated and statistical analysis was conducted in R (Version 4.0.3; R Core Team, 2020). Univariate descriptive statistics was used for sociodemographic variables. Continuous variables are presented as means and standard deviations, and categorical variables are presented as frequencies and percentages.

The Cronbach's alpha coefficient was used to measure internal consistency of the COHIP-SF 19. As the Kolmogorov-Smirnov test indicated, the COHIP-SF-19 did not follow a normal distribution. We determined the magnitude of change in OHRQoL after dental treatment by subtracting the COHIP-SF 19 scores at follow-up from those at baseline. The Wilcoxon signed-rank test served to compare baseline and follow-up scores regarding statistical significance of potential changes. Significance level was set at 5% (0.05).

The effect size was calculated by dividing the mean of change score by the standard deviation of the baseline score. An effect size of <0.2 indicated a small but clinically meaningful magnitude of change, 0.3–0.7 a moderate change, and >0.7 a large change.

## Results

The sociodemographic characteristics of the studied population are described in Table 1. Eighty children participated in the study, 39 boys (48.8%) and 41 girls (51.3%), mean age 9.4 ± 1.0. Regarding province of residence, 52 schoolchildren (65%) lived in San José, the capital city of Costa Rica, followed by eight schoolchildren (10%) who lived in Alajuela, 13 schoolchildren (16.3%) who lived in Cartago, six schoolchildren (7.5%) who resided in Heredia, and only one (1.3%) who lived in Limón. Regarding monthly household income, the minimum income reported was one hundred thousand colones (approximately $170), the maximum reported was two hundred thousand colones (approximately $340), and the average was five hundred forty-one thousand colones (approximately $916). Thus, according to Costa Rica's Home Mortgage Bank (BANHVI), 56.3% of the studied population belonged to a low-income family, 41.2% to a middle-income family, and 2.5% to a high-income family.

**Table 1:** Sociodemographic characteristics of the sample.

Patient variable	** **
Mean age	9.4 ± 1.0 years
Gender	***n* (%)**
Male	39 (48.8)
Female	41 (51.3)
Province	***n* (%)**
San José	52 (65.0)
Alajuela	8 (10.0)
Cartago	13 (16.3)
Heredia	6 (7.5)
Limón	1 (1.3)
Mean income	₡541,000
Low income	56.3%
Middle income	41.2%
High income	2.5%
Family structure	***n* (%)**
Together	47 (58.8)
Separated	32 (40.0)
Mother's education	***n* (%)**
Incomplete primary	5 (6.3)
Complete primary	9 (11.3)
Incomplete secondary	27 (33.8)
Complete secondary	17 (21.3)
Incomplete university	3 (3.8)
Complete university	19 (23.8)
Father's education	***n* (%)**
Incomplete primary	3 (3.8)
Complete primary	16 (20.0)
Incomplete secondary	22 (27.5)
Complete secondary	16 (20.0)
Incomplete university	5 (6.3)
Complete university	15 (18.8)
Type of house	***n* (%)**
Own	43 (53.8)
Rented	29 (36.3)
Borrowed	8 (10.0)
Mean inhabitants living in the same house	4.4 ± 1.3

*n*, number; %, percentage.

₡, Costa Rica's currency, colones.

In relation to family structure and stability, 58.8% of the children's parents lived together, either married or free union, while 40.0% of the children's parents were separated and lived in a different household. One participant preferred not to answer this question. In addition, when parents were asked about household owning or renting, 43 (53.8%) answered owning their house, 29 (36.3%) lived in a rented house, and eight (10.0%) lived in a borrowed household. Moreover, in relation to the number of family members living in the same household as the patient, a minimum of two people, a maximum of nine, and an average of four people was reported.

Regarding the mothers' educational level, 11.3% completed primary school, 21.3% completed secondary school, and 23.3% completed university studies. Additionally, for the father's educational level, 20.0% completed primary school, 20.0% completed high school, and 18.8% completed university studies. Of the mothers, 6.3% did not complete primary education, 33.8% did not conclude secondary studies, and 23.8% did not finish university studies. Furthermore, 3.8% of the children's fathers did not complete primary school, 27.5% did not complete secondary school, and 18.8% did not finish university studies (Table 1). Three of the surveyed parents did not answer when asked about their educational level.

The schoolchildren who participated in this study were treated for caries, hypomineralizations (including molar incisor hypomineralizations, amelogenesis imperfecta, and fluorosis), and dental malocclusions. The prevalence of dental caries was 56.1% with a mean deft and DMFT score of 3.15 ± 0.96 and 2.22 ± 0.77, respectively. Prevalence of hypomineralizations was 53.7% and dental malocclusions was 82.9%. Treatment of hypomineralizations included application of fluoride varnish in 96% of participants, placement of conventional glass ionomer in 2% of the participants, and, in severely damaged teeth, the use of stainless-steel crowns in 2% of the participants. All patients with dental malocclusions presented Class I by angle classification and were treated with different orthodontic appliances, including retainers, appliances for arch development, or fixed appliances. Treatment period of malocclusions varied between patients, from 8 to 10 months, depending on the individualized treatment plan. The Simplified Oral Hygiene Index was 1.45 ± 0.45 pre-treatment and 1.42 ± 0.43 post-treatment (*p *> 0.05). Both scores are considered as a fair level of oral hygiene (Table 2).

**Table 2:** Oral conditions in the studied population.

Oral conditions	Prevalence
Caries	56.1%
Hypomineralizations	53.7%
Malocclusions	82.9%
Indexes	Mean scores (SD)
Deft	3.15 (±0.96)
DMFT	2.22 (±0.77)
OHI-S	
Before dental treatment	1.45 (±0.45)
After dental treatment	1.42 (±0.43)

%, Percentage; SD, standard deviation.

Deft, primary teeth: d, decayed, e, extracted due to caries, f, filled, t, teeth.

DMFT, permanent teeth: Decayed, M, missing due to caries, F, filled, T, teeth.

OHI-S, simplified oral hygiene index.

Cronbach's alpha coefficient before treatment was 0.63 and after treatment was 0.68, indicating the questionnaire had an acceptable level of internal reliability. Gender or age did not play a significant role in pre- and post-treatment scores of the COHIP-SF-19 or in improvement level of quality of life (both *p*s > 0.05). Table 3 summarizes the frequency distribution of participants’ responses to COHIP-SF-19 components before dental treatment. Of the Oral Health Well-Being components of COHIP-SF-19, bad breath at 97.5% and gingival bleeding at 85.0% were the most reported. From the functional problems experienced in the previous three months, difficulty saying certain words at 71.2% was the most reported, followed by difficulty eating liked foods at 67.5%. Regarding social/emotional impacts experienced before dental treatment, 89.0% of the schoolchildren felt they looked different and 81.2% felt worried and/or anxious. About the impact on school or environmental domain, 87.5% of schoolchildren missed school, and 51.2% of schoolchildren did not want to speak/read out loud in class. For impact on self-image domain, 7.5% of schoolchildren did not feel confident ever and 10.0% of schoolchildren felt they were not attractive.

**Table 3:** Frequency distribution (%) of the responses for child oral health impact profile-short form 19 (COHIP-SF-19) items before dental treatment (*n* = 80).

COHIP-SF-19 items	0 *n* (%)	1 *n* (%)	2 *n* (%)	3 *n* (%)	4 *n* (%)	Total
**Domain 1: Oral Health Well-being**						
Q1. Had pain in your teeth/toothache	18.8	10.0	46.3	23.8	1.3	100.0
Q2. Had discolored teeth or spaces between your teeth	25.0	16.3	35.0	21.3	2.5	100.0
Q3. Had crooked teeth or spaces between your teeth	25.0	16.3	35.0	21.3	2.5	100.0
Q4. Had bad breath	2.5	7.5	25.0	52.5	12.5	100.0
Q5. Had bleeding gums	15.0	13.8	25.0	33.8	12.5	100.0
**Domain 2: Functional Well-being**						
Q9. Had difficulty eating food you like	32.5	13.8	25.0	20.0	8.8	100.0
Q12. Had trouble sleeping	61.3	10.0	18.8	10.0	0.0	100.0
Q15. Had difficulty saying certain words	28.8	15.0	38.8	12.5	5.0	100.0
Q19. Had difficulty keeping your teeth clean	0.0	0.0	10.0	16.3	73.8	100.0
**Domain 3: Social/Emotional Well-being**						
Q6. Been unhappy or sad	36.3	12.5	30.0	12.5	8.8	100.0
Q10. Felt worried and/or anxious	18.8	7.5	31.3	26.3	16.3	100.0
Q11. Avoided smiling or laughing with other children	55.0	11.3	15.0	5.0	13.8	100.0
Q14. Teased, bullied, or called names by other children	88.8	3.8	5.0	1.3	1.3	100.0
Q16. Felt that you look different	11.3	10.0	51.3	15.0	12.5	100.0
Q18. Been worried about what people think of you	52.5	6.3	23.8	8.8	8.8	100.0
**Domain 4: School Environment**						
Q7. Missed school for any reason	12.5	5.0	28.8	38.8	15.0	100.0
Q13. Did not want to speak/read out loud in class	48.8	11.3	31.3	7.5	1.3	100.0
**Domain 5: Self-Image**						
Q8. Been confident	7.5	17.5	42.5	27.5	5.0	100.0
Q17. Felt that you were attractive	10.0	16.3	45.0	21.3	7.5	100.0

N, number; %, percentage; Q, question. Scale ranging from “never” = 0, “almost never” = 1, “sometimes” = 2, “fairly often” = 3, and “almost all the time” = 4. The number after Q means the order that the question appeared in the COHIP-19 questionnaire. Each question belongs to a specific domain.

Table 4 shows how the frequency distribution of the responses for COHIP-SF-19 items changed after dental treatment in the studied population. With reference to Oral Health Well-being, most of the studied population, 96.2%, reported feeling no pain or almost no pain in their teeth. Even though scores lessened post-treatment, major concerns revealed by schoolchildren were still bad breath in 71.2% of the studied population and bleeding gums in 63.7%. In response to Functional Well-being after treatment, 81.2% said they had difficulty keeping their teeth clean. The scores post-treatment for difficulty saying certain words decreased, as did scores for difficulty eating liked foods, which were pre-treatment preoccupations expressed by schoolchildren. As for Social/Emotional impacts experienced after dental treatment, 77.5% felt they looked different and 63.7% expressed feeling worried or anxious. About the impact on school or environmental domain, 48.7% of schoolchildren missed school, and 41.2% of schoolchildren did not want to speak/read out loud in class. After treatment, impact on self-image domain resulted in 37.5% of schoolchildren not feeling confident ever and the same percentage of schoolchildren as before felt they were not attractive.

**Table 4:** Frequency distribution (%) of the responses for child oral health impact profile-short form 19 (COHIP-SF-19) items after dental treatment (*n* = 80).

COHIP-SF-19 items	0 *n* (%)	1 *n* (%)	2 *n* (%)	3 *n* (%)	4 *n* (%)	Total
**Domain 1: Oral Health Well-being**						
Q1. Had pain in your teeth/toothache	57.4	38.8	3.8	0	0	100.0
Q2. Had discolored teeth or spaces between your teeth	20.0	63.8	15.0	1.3	0.0	100.0
Q3. Had crooked teeth or spaces between your teeth	71.3	22.5	5.0	1.3	0.0	100.0
Q4. Had bad breath	28.8	57.5	12.5	1.3	0.0	100.0
Q5. Had bleeding gums	36.3	53.8	10.0	0.0	0.0	100.0
**Domain 2: Functional Well-being**						
Q9. Had difficulty eating foods you like	47.5	50.0	2.5	0.0	0.0	100.0
Q12. Had trouble sleeping	87.5	12.5	0.0	0.0	0.0	100.0
Q15. Had difficulty saying certain words	56.3	36.3	7.5	0.0	0.0	100.0
Q19. Had difficulty keeping your teeth clean	18.8	26.3	33.8	13.8	7.5	100.0
**Domain 3: Social/Emotional Well-being**						
Q6. Been unhappy or sad	61.3	31.3	7.5	0.0	0.0	100.0
Q10. Felt worried and/or anxious	36.3	45.0	16.3	2.5	0.0	100.0
Q11. Avoided smiling or laughing with other children	85.0	10.0	5.0	0.0	0.0	100.0
Q14. Teased, bullied, or called names by other children	100.0	0.0	0.0	0.0	0.0	100.0
Q16. Felt that you look different	22.5	26.3	48.8	1.3	1.3	100.0
Q18. Been worried about what people think of you	57.5	28.8	13.8	0.0	0.0	100.0
**Domain 4: School Environment**						
Q7. Missed school for any reason	51.3	46.3	2.5	0.0	0.0	100.0
Q13. Did not want to speak/read out loud in class	58.8	33.8	7.5	0.0	0.0	100.0
**Domain 5: Self-Image**						
Q8. Been confident	37.5	43.8	8.8	5.0	5.0	100.0
Q17. Felt that you were attractive	37.5	43.8	15.0	0.0	3.8	100.0

n, number, %, percentage; Q, question. Scale ranging from “never” = 0, “almost never” = 1, “sometimes” = 2, “fairly often” = 3, and “almost all the time” = 4. The number after Q means the order that the question appeared in the COHIP-19 questionnaire. Each question belongs to a specific domain.

Table 5 shows the descriptive statistics of the sum of scores pre- and post-dental treatment of the COHIP-SF-19.

**Table 5:** Descriptive statistics of the sum of scores before and after dental treatment of the child oral health impact profile-short form 19 (COHIP-SF-19) (*n* = 80).

	Sum of scores before dental treatment	Sum of scores after dental treatment
Mean	53.7	31.5
Variance	60.5	17.3
Standard Deviation	7.8	4.2
Median	54.5	31.0
Minimum	35.0	22.0
Maximum	79.0	41.0
95% Confidence interval of the mean, lower bound	52.0	31.0
95% Confidence interval of the mean upper bound	55.4	32.4

Table 6 describes COHIP-SF-19 total score and domain scores before and after dental treatment. The overall COHIP-SF-19 score decreased from 53.7 ± 7.8 to 31.5 ± 4.2 after treatment (*p* < 0.001; *d* = 3.5). All COHIP-19 domain scores were significantly and clinically improved at post-treatment (all *p*'s < 0.001 and effect size = 3.3, 1.6, 2.1, 1.2 and 1.5, respectively). In regard to the first domain, Oral Health Well-being, tooth discoloration had the highest scores, both before and after dental treatment, 4.4 ± 0.9 and 2.0 ± 0.6, respectively. This concern improved post-treatment (*d* = 3.1). The highest score before dental treatment in the second domain evaluated, Functional Well-being, was “trouble sleeping due to teeth/face”, 3.4 ± 1.2. These scores lowered after dental treatment to 1.5 ± 0.6 (*d* = 2.0). Relating to the third domain evaluated, Social/Emotional Well-being, before dental treatment, the greatest score was on being unhappy or sad, 3.1 ± 1.3, followed by being teased, bullied, or called names by other children, 3.0 ± 1.0. Both scores decreased post-treatment to 1.9 ± 0.8 and 1.9 ± 0.9 (*d* = 1.1 and *d* = 1.2, respectively). About the fourth domain, School Environment, before treatment, the greatest score was on the item “missed school for any reason” 3.1 ± 1.1. After receiving dental treatment, the score decreased to 2.3 ± 0.9 (*d* = 0.8), which shows an improvement in the patient's daily activities. And finally, domain five, Self-image, reports the greatest score before dental treatment was the item related to confidence, 4.6 ± 0.7, and after treatment there was an improvement in the score, 2.6 ± 1.2 (*d *= 2.0).

**Table 6:** Mean values of child oral health impact profile-short form 19 (COHIP-SF-19) scores before and after dental treatment.

	Before treatment	After treatment	Effect size^a^	*p* value
	Mean (SD)	Mean (SD)		
COHIP-SF-19 Overall score	53.7 (7.8)	31.5 (4.2)	3.5	<0.001*
Domain 1: Oral Health Well-being	16.6 (2.9)	8.4 (1.9)	3.3	<0.001*
Pain/toothache	2.8 (1.1)	1.5 (0.6)	1.5	
Had discolored teeth or stains on the teeth	4.4 (0.9)	2.0 (0.6)	3.1	
Crooked teeth or spaces	2.6 (1.2)	1.4 (0.6)	1.3	
Bad breath	3.6 (0.9)	1.9 (0.7)	2.1	
Bleeding gums	3.1 (1.3)	1.7 (0.6)	1.4	
Domain 2: Functional well-being	9.8 (2.9)	6.2 (1.5)	1.6	<0.001*
Trouble chewing firm foods/Difficulty eating	2.5 (1.3)	1.5 (0.6)	1.0	
Trouble sleeping due to teeth/face	3.4 (1.2)	1.5 (0.6)	2.0	
Difficulty saying certain words	3.0 (1.0)	2.0 (1.1)	0.8	
Difficulty keeping teeth clean	2.1 (1.4)	1.6 (0.7)	0.4	
Domain 3: Social/Emotional Well-being	16.5 (3.2)	10.8 (2.1)	2.1	<0.001*
Been unhappy or sad	3.1 (1.3)	1.9 (0.8)	1.1	
Felt worried or anxious	2.0 (1.1)	1.5 (0.6)	0.6	
Avoided smiling or laughing with other children	2.1 (1.5)	1.2 (0.5)	0.8	
Felt that you looked different	1.8 (1.1)	1.1 (0.3)	0.9	
Been worried about what other people think about your teeth, mouth, or face	1.2 (0.7)	1.0 (0.0)	NA	** **
Been teased, bullied, or called names by other children	3.0 (1.0)	1.9 (0.9)	1.2	** **
Domain 4: School environment	5.6 (1.7)	3.8 (1.2)	1.2	<0.001*
Missed school for any reason	3.1 (1.1)	2.3 (0.9)	0.8	** **
Not wanted to speak/read out loud in class	2.5 (1.2)	1.5 (0.6)	1.1	** **
Domain 5: Self-image	6.8 (1.5)	4.2 (1.4)	1.5	<0.001*
Been confident	4.6 (0.7)	2.6 (1.2)	2.0	** **
Felt that you were attractive	2.1 (1.4)	1.6 (0.7)	0.5	** **

Wilcoxon's signed rank test.

* Significant at *α* = 0.05 level.

^a^ Calculated using Cohen's *d* (=difference/SD): an effect size of 0.2 < *d* ≤ 0.5 was considered small, 0.5 < *d* ≤ 0.8 was considered intermediate, and *d* > 0.8 was considered large.

## Discussion

The findings of our study highlight the magnitude and impact of treatment of caries, hypomineralizations, and dental malocclusions in OHRQoL in schoolchildren aged 8–12 years. There is a paucity of studies on oral health conditions in Costa Rican schoolchildren. We found a high prevalence of caries; the deft/DMFT was 3.15/2.22. Most hypomineralizations were managed conservatively by applying fluoride varnish, suggesting even minimally invasive procedures have a positive effect in OHRQoL. None of the recruited participants presented skeletal class II or III jaw relationships. These patients had Class I malocclusions by Angle classification. Our results demonstrate a positive impact in OHRQoL concerning early orthodontic treatment among children in the mixed or initial permanent dentition phase.

We selected this age range, 8–12 year olds, since studies evaluating OHRQoL in this age population are scarce. Children aged 8–12 are suitable for identifying oral health situations in mixed dentition. In addition, the COHIP-SF-19 questionnaire is suitable for ages between 8 and 15. The instrument has good psychometric properties to measure OHRQoL across school-age pediatric populations (10).

We characterized some sociodemographic and socio-economic variables that have been found to influence OHRQoL in children. According to the sociodemographic data collected, most of the children were from the capital city of Costa Rica, the province where most economic activities take place. Most parents reported living in owned households. Regarding educational assessment, many of the parents did not complete secondary school. This data coincides with The Organization for Economic Co-operation and Development review of the labor market and social policies in Costa Rica, which states that more than half of the adult population in the country has attained less than upper-secondary education (42). Kragt et al., evidenced that children of fathers and mothers with low educational level have lower OHRQoL than children of fathers with a high educational level (43). In the same line, these authors reported significantly lower OHRQoL in children of unemployed fathers, children with a low household income, or a single-parent family. In our study, the average economic income of the parents oscillated around five hundred forty-one thousand colones (approximately $916), which is above the minimum base salary in the country. A systematic review and meta-analysis provided information on four cross-sectional studies carried out in upper-middle countries, like Costa Rica, and found that children from low-income families were more likely to have poor OHRQoL (32).

Although one of our limitations was that we did not perform further analysis on the association between sociodemographic and socioeconomic disparities and COHIP-SF-19 responses, scientific literature confirms that socioeconomic variables interfere in children's condition of life (3, 29, 33). Thus, children with a lower family socioeconomic position perceive a lower OHRQoL. This perception is independent of their clinical oral health status. It is believed children who live with families with higher incomes can have better oral hygiene behaviors and better access to preventive and health care interventions, resulting in a better quality of life.

Children's family environment could also be linked to OHRQoL. A study by Ahuja & Ahuja recognized that children living with both parents reported better OHRQoL as compared to single parents or guardians (44). This may be explained by the theory that economic resources and parental care are commonly shared among siblings. In our study, most of the participants lived with both biological parents and the mean number of inhabitants in the same household was four. This fourth person could be another sibling or another member of the family.

We found significant improvements in the perceived oral health of patients after receiving dental care, which further supports the notion that oral health is an important aspect in the quality of life of children. The COHIP-SF-19 scores of schoolchildren were significantly and clinically improved after dental treatment. These results agreed with those of previous studies that reported reduced OHRQoL due to dental caries (45, 46), hypomineralizations (47, 48), and dental malocclusions (49). In these studies, OHRQoL improved after treatment (45, 46, 48–50). Even though these previous studies were performed using different OHRQoL questionnaires, and were applied to different age ranges, statistical and clinical improvements were found in all subscales, including oral symptoms and functional problems.

COHIP-SF-19 Oral Health Well-being domain incorporates specific oral health symptoms (pain, spots on teeth, spaces between teeth, bad breath, and bleeding gums). In this study, the most evident improvement in the Oral Health Well-being domain was tooth discoloration, which coincides with a study on the effects of dental treatment and systemic disease on OHRQoL in Korean pediatric patients (51). Other major concerns in the Oral Health Well-being domain, before and after dental treatment, were bad breath and gingival bleeding. Even though we did not assess gingival status in these patients, there may be two possible reasons for these concerns: first, all participants reported at baseline a difficulty in keeping their teeth clean, and the simplified oral hygiene index did not change significantly after treatment in these patients. Therefore, oral hygiene instructions need to be reinforced during dental care in our clinical setting. Second, during puberty, children are more susceptible to gingivitis. Ahn et al., found the items that generated the greatest discomfort in 8–15-year-old schoolchildren were food sticking in the teeth, crooked teeth, spaces between teeth, and difficulty in maintaining oral hygiene (52). Similarly, these concerns were reported by our participants pre-treatment and scores improved significantly after dental treatment.

Functional Well-being relates to items pertaining to the child's ability to do everyday tasks or activities. Concerning this domain, the most prominent change was in the item “trouble sleeping due to teeth/face”. A high percentage of the children interviewed, 81.2%, revealed having dental pain pre- dental treatment. Dental pain can affect oral function for children, along with eating and sleeping. A study by Moro et al., concluded that self-reported trouble sleeping due to dental problems in children is associated with untreated dental caries (53). Since sleep has a significant impact on children's learning, memory, and school performance, professionals should pay special attention to preventing and treating oral conditions that can disturb the quality of sleep.

Social/Emotional Well-being includes mood states and interactions with peers. Various dental treatments have been associated with an improvement in OHRQoL in children of different ages in different populations, and this improvement is not only attributed to pain and improved function but also to fostering social interactions (54). In the current study, the greatest effect size post-treatment was on the item “been unhappy or sad”. This is not surprising since previous findings suggest poor oral health among children comprises not only their functional limitations but also their social and emotional well-being (55). Honkala et al., showed that being happy was an important predictor of better oral hygiene habits in 11–13 year olds (56). Also, children with cavitated lesions are more likely to be worried about their oral health status and caries can affect certain emotional aspects among adolescents (57, 58). Tuchtenhagen et al. (59) found the presence of untreated dental caries and malocclusions was associated with lower levels of happiness and played important roles in social interaction and acceptance.

Referring to School Environment, a high percentage of the studied population reported missing school due to problems related with their teeth, mouth, or face before treatment. This changed significantly after treatment. Research has shown that children with poorer oral health status were more likely to experience dental pain, and consequently miss school (60). At pre-treatment, most schoolchildren expressed they did not want to speak or read out loud in class. However, children were less apprehensive post-treatment. On the contrary, Anthony et al., studied the impact on malocclusion on OHRQoL in early adolescents in Ndola, Zambia (61). Impact on the School Environment domain was reported by only 2.9% of participants, and 7% of children did not want to speak/read out loud in class. Based on our results, we suggest improving child's oral health status, since it may be a method to enhance their learning experience at school.

Self-image comprises positive feelings about oneself. Although the Self-image domain improved after dental treatment in our study, we found some children had a negative perspective about their self-confidence and attractiveness after dental treatment. In accordance with our findings, validation studies of the COHIP-SF-19 have reported these two items were the most unanswered questions. It has been suggested that these two items are the only positively worded questions, which could potentially confuse participants (62).

Like previous studies, more girls sought dental treatment than boys during the recruitment period of our investigation. However, in this study, gender and age were not important factors in OHRQoL, in agreement with a similar study performed by Mendonça et al. (63). These authors assessed the impact of dental treatment on OHRQoL in 6–8-year-old Brazilian schoolchildren. Even though Mendonça et al. used a different instrument to assess OHRQoL, they found a similar mean score change in the treatment group (overall score 15.44 ± 13.28). Our mean total change in the COHIP-SF-19 was 22.2 ± 3.6. In our study, and the latter investigation, the effect size and differences among means before and after dental treatment were considered large when comparing phase one, before treatment, and phase two, after treatment. Likewise, Vollú et al., who evaluated OHRQoL thirty days after dental treatment in 16 preschool children, reported a great change (effect size >0.7) after treatment in all domains except for child self-image and social interaction (64). Overall, our data confirms that quality of life improves after dental treatment in children. OHRQoL complements clinical data and may be useful to evaluate efficacy of dental care provided.

## Conclusion

This study affirms that dental caries, hypomineralizations, and dental malocclusions have a negative impact on the quality of life of 8–12-year-old schoolchildren. Post-treatment OHRQoL values were significantly lower and might be associated with the resolution of pre-treatment symptoms or difficulties in having self-confidence. The family's sociodemographic characteristics unlikely changed during the study period. Thus, it appears that dental treatment *per se* improves self-perceptions and quality of life in children in the short term.

One of the strengths of our study is the excellent follow-up response rate; the response rate was 100% in the one-month follow-up after treatment. Furthermore, the instrument used in this study has been validated in Spanish and has shown good psychometric properties. Measures of internal consistency, effect size, and mean score differences demonstrated that this questionnaire is valid for evaluating changes in OHRQoL of children aged 8–12 years after dental treatment. Therefore, this instrument could be used in future clinical trials in this age group.

The limitations of our study include the short-term evaluation of OHRQoL after dental treatment was completed. Greater evaluation periods have been recommended to evaluate long-term effects on OHRQoL. We plan to follow-up on these patients to demonstrate if the effect of dental treatment is sustainable over time or not. It would be interesting to determine satisfaction and long-term OHRQoL, since oral health needs in this population may change with permanent dentition.

## Data Availability

The raw data supporting the conclusions of this article will be made available by the authors, without undue reservation.
